# Understanding contextual barriers, supports, and opportunities for physical activity among Mexican-origin children in Texas border *colonias*: A descriptive study

**DOI:** 10.1186/1471-2458-13-14

**Published:** 2013-01-08

**Authors:** M Renée Umstattd Meyer, Joseph R Sharkey, Megan S Patterson, Wesley R Dean

**Affiliations:** 1 Baylor University, Department of Health, Human Performance, and Recreation, One Bear Place 97313, Waco, TX, 76798, USA; 2 Program for Research in Nutrition and Health Disparities, Department of Health Promotion and Community Health Sciences, Texas A&M School of Rural Public Health, TAMU, 1266, College Station, TX, 77843-1266, USA

**Keywords:** Mexican-origin children, physical activity, *colonias*, promotora-researchers, environmental support.

## Abstract

**Background:**

The increasing numbers of *colonias* along the U.S.-Mexico border are characterized by disproportionately poor families of Mexican-origin, limited access to resources and health services, and heightened risk for obesity and diabetes. Despite consistent evidence supporting physical activity (PA) in prevention of chronic diseases, many individuals of Mexican-origin, including children, fail to meet PA recommendations. Environmental influences on PA, founded in ecological and social cognitive perspectives, have not been examined among children living in *colonias*. The purpose of this study was to identify and better understand (1) household and neighborhood environmental PA resources/supports, (2) perceived barriers to engaging in PA, and (3) PA offerings, locations, and transportation characteristics for Mexican-origin children living in *colonias*.

**Methods:**

Data for this study were collected by *promotora*-researchers (indigenous community health workers trained in research methods) using face-to-face interviews conducted in Spanish. The sample consists of 94 mother-child dyads from Texas border *colonias* in Hidalgo County. Interviews included questionnaire items addressing PA barriers, household and neighborhood environmental support assessments conducted with each dyad, and open-ended questions that were coded to identify availability and locations of PA opportunities and transportation options. Descriptive statistics were calculated and differences between genders, birth countries, and BMI categories of children were determined using chi-square tests.

**Results:**

All children were of Mexican-origin. The most frequently reported barriers were unleashed dogs in the street, heat, bad weather, traffic, no streetlights, and no place like a park to exercise. Prominent locations for current PA included schools, home, and parks. Common PA options for children were exercise equipment, running, playing, and sports. Environmental assessments identified exercise equipment (bicycles/tricycles, balls, etc.…), paved/good streets, yard/patio space, and social norms as the most frequent household or neighborhood resources within these *colonias*. Differences in PA barriers, options, and environmental resources for genders, birth countries, and BMI categories were detected.

**Conclusions:**

This study suggests that PA environmental resources, barriers, and opportunities for *colonias* children are similar to previous studies and distinctively unique. As expected, built resources in these communities are limited and barriers exist; however, knowledge of PA opportunities and available PA resources within *colonias* households and neighborhoods offers insight to help guide future research, policy, and PA initiatives.

## Background

Obesity, diabetes, related health conditions, and associated health burden disproportionately affect underserved, marginalized populations.[1-3] While the role of physical activity in prevention of obesity, diabetes, and related chronic conditions is well established,[4,5] these same populations face greater disparity in access to health care and health promotion services, including physical activity facilities and programming.[5,6] One underserved population characterized by both disproportionate ethnicity-related and socioeconomic burden is Mexican-origin families residing in impoverished *colonias* in the Lower Rio Grande Valley along the Texas-Mexico border.[6,7] The Mexican-origin population is the fastest growing subpopulation in the U.S., with the majority of this population growth occurring among the growing number of *colonias* along the U.S. border with Mexico, especially in Texas, and in new-destination immigrant communities throughout the nation.[8,9]

**Figure 1:**
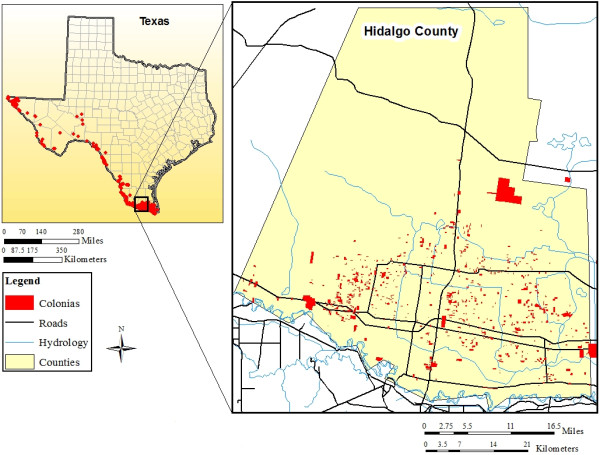
***Colonias *along the Texas-Mexico border and in Hidalgo County.** This map was developed using mapped-data from the Colonias Reader (Ed.s Donelson and Esparza). Geospatial boundaries for *colonias* in the State of Texas were downloaded from the U.S. Geological Survey’s Border Environmental Health Initiative (BEHI) website: http://borderhealth.cr.usgs.gov/datalayers.html. The map was created courtesy of Laura M. Norman, USGS Research Physical Scientist, July 30, 2012.

**Figure 2:**
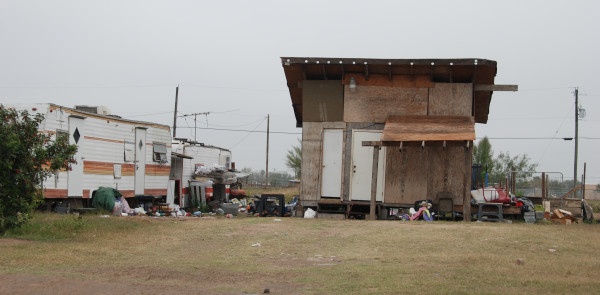
***Colonia *residences in Hidalgo County.** Images were taken January 2011.

*Colonias* (Spanish for neighborhood or community) are substandard residential areas that usually lack infrastructure, exist along the U.S.-Mexico border, and were originally developed from subdivided agricultural lands in response to a deficit in low-income housing.[6,10]*Colonias* are characterized by inadequate (sometimes unpaved) roads, variable types and conditions of housing ranging from trailers to self-built houses, and often lack safe water, sewer services, and electricity.[6,11,12] The U.S. Government has defined *colonias* in various government and water codes as economically distressed communities consisting of low or very-low income households based on the Federal poverty index, with inadequate water or waste water services, located at or near the U.S.-Mexico border area with an outer range stretching from 50–150 miles into the U.S.[12,13] To provide a visualization, *colonias* have been described as Third World areas even though they are located within the United States of America.[10] Over 2,500 *colonias* span the U.S.-Mexico border, with approximately 2,200 providing homes to more than 400,000 in Texas. Within Texas, over 860 *colonias*, with an estimated 156,132 residents, are located in Hidalgo County alone. Figure 1 identifies *colonias* along the Texas-Mexico border and Hidalgo County. In addition, the border population is growing at a rate nearly double that of the rest of Texas, most of which is occurring in *colonias.*[10] Approximately 97% of those residing in *colonias* are of Hispanic origin (2/3 U.S. born), with an estimated 37% lacking spoken English proficiency.[14] The image in Figure 2 provides an example of typical *colonia* residences in the south Texas Lower Rio Grande Valley.

### Texas colonia residents

It is well documented that obesity and diabetes rates are astoundingly high across the nation and are even greater for Mexican-Americans, who have an 87% greater chance of diabetes diagnosis as compared to non-Hispanic whites. Rates along the U.S.-Mexico border are often even more extreme.[5,15-19] Given this stark health disparity, diabetes was recently identified as the prevailing preventable health issue among *colonias* residents.[6] However, this health disparity is further accentuated when considering additional health and economic disparities. Relationships between diabetes and obesity/overweight, hypertension, and high cholesterol are supported,[20] for which Mexican-American adults and children are at escalated risk of diagnosis, especially those living in *colonias* and other new-destination immigrant communities.[5,16,18,21-23] Evidence also suggests these rates continue to increase disproportionately for the poorest in our nation, including Mexican-Americans.[7,18,19] Mexican-Americans residing in *colonias* along the Texas-Mexico border unfortunately epitomize this risk given their markedly low household income rates (estimated at < $834/month), consistent designation of persistent poverty, and high unemployment rates (20-70% as compared to 7% nationally).[6,24]

While residents of *colonias* face numerous health and economic disparities, it is important to focus and capitalize on the communities’ cultural assets and wealth.[25-28] Cultural wealth demonstrated in minority communities like *colonias* include familial capital,[29,30] meaning that kinship ties to extended family and neighbors allow residents to learn the importance of maintaining a healthy connection to the community and its resources, and social capital,[31] which are peer and other social contacts that can provide both instrumental and emotional support to navigate through society.[25] The cultural strengths of *colonias* are valuable when considering an ecological approach to behavior change.

### The Role of physical activity

Epidemiological data suggest a heightened and serious health risk for the burgeoning *colonia* population. Evidence consistently supports a positive and significant relationship between physical activity participation and reduced risks for diabetes, obesity/overweight, hypertension, and high cholesterol across all people and age groups.[4,5] However, few Mexican-Americans and lower-income Americans, including children, report engaging in physical activity at levels related with health benefits.[32,33] Of adult U.S. Hispanics, 57.9% report not engaging in sufficient physical activity to experience health benefits.[32] Among Hispanic high school youth in 2009, only 39.3% reported participating in 60 minutes of physical activity on 5 or more of the previous days, as compared to 46.6% of the entire high school population and 54.6% of non-Hispanic white youth.[34] Based on the consistent relationship between physical activity participation and the prevention of diabetes and obesity, insight is urgently needed to better understand how to support and increase physical activity participation among this growing and disproportionately high risk population.

*Environmental Influence:* While substantial research exists examining intrapersonal-level factors that influence physical activity, recently researchers have also begun to establish a strong evidence base regarding relationships between physical activity and the physical environment.[35,36] Yet, as with other health issues, minorities, immigrant populations, and *colonia* residents in particular are rarely examined.[6,37] Current evidence describes the influence of physical environmental features, such as street design, network connectivity, site design, density, safety, lighting, and aesthetic appeal of neighborhoods as important factors related with physical activity participation.[38] With the common absence of connectivity, playgrounds, parks, traditional yards, safe open spaces, and recreation facilities in *colonias*, physical activity could be severely limited as it is understood in urban-suburban and even rural literature, especially among youth.[6] However, these physical features and environmental concepts may not play significant roles, or may play distinctly unique roles in understanding physical activity participation among children residing in *colonias*.[6] Examining and understanding the physical environment in regards to physical activity engagement in *colonia* communities is vital to development of effective, efficient, and safe physical activity initiatives and policies for these underserved communities.

Two primary ways environmental influences are assessed are through objective measurement (audits/assessments/observations) to capture the physical or built environment and self-report measures to understand perceived environmental supports and barriers.[39] Both concepts of environment, the actual physical environment and the perceived environment, are supported theoretically through ecological and social cognitive approaches. Ecological and social cognitive approaches are premised on multiple levels of reciprocal behavioral influence, where individual perceptions and beliefs are distinct, yet related and influenced by, the actual environment.[35,38,40,41] Given these theoretical pretenses, understanding both actual and perceived environmental factors is important, as either could facilitate or hinder physical activity participation.

Culturally-sensitive research, especially among hard-to-reach populations, requires knowledge of the study population and trust of researchers by participants.[42] Research supports the importance of collaborating with trained *promotoras* who become trusted members of the research team while also serving as cultural brokers, having knowledge of the values, beliefs, and practices within Mexican-American households in the *colonias*.[43-45] Programs using community health workers (*promotoras*) have been some of the most successful in delivering primary and preventative health services and information in Mexican-American communities and specifically *colonias*.[6,45-49]

The purpose of this study was to identify and better understand (1) household and neighborhood environmental physical activity resources/supports, (2) perceived barriers to engaging in physical activity, and (3) physical activity offerings, locations, and transportation characteristics for Mexican-origin children living in *colonias*. Given potential differences in physical activity participation and perceived barriers and environmental supports between female and male children, those children born in Mexico and the U.S., and children with different weight status, [50-54] a secondary aim of this study was to examine differences in physical activity resources and perceived barriers to physical activity between these groups.

## Methods

Mother-child dyads (n=94) were recruited by *promotora*-researchers (indigenous community health workers trained in research methods) to facilitate and partner within a project funded to examine food availability among Mexican American families in Texas colonias.[11,55-57] Results from this study have been reported elsewhere.[11,55-57] Mother-child dyads consisted of a Mexican-origin mother and one of her children aged 6–11 who were living in *colonias* located in one of four large areas of *colonias* in Hidalgo County, Texas. These four areas were selected by team *promotora*-researchers based on prior research and outreach activities. Census block groups were used to spatially select 56 unique *colonias,* each varying in geography and population. Family dyads were subsequently recruited from these 56 *colonias*.

The present study used a mixed-methods approach, employing both quantitative and qualitative assessments. Measures were selected and refined during the winter of 2010 and spring of 2011 and data collection occurred during the spring of 2011. Interview items were designed to identify perceived household and neighborhood activity-supportive resources and environmental barriers pertaining to child physical activity, as well as current physical activity opportunities, locations, and transportation availability for *colonias* children. Environmental assessments were designed to identify activity-supportive resources within *colonias* households and neighborhoods. *Promotora*-researchers completed training in data collection and protection of participant confidentiality as provided by the project’s investigators prior to data collection time periods. [57]*Promotora*-researchers also provided feedback regarding item wording, translation, understandability, and cultural appropriateness of all items. The trained *promotora*-researchers who were part of this research team are trusted by the *colonia* residents and served as cultural brokers, having knowledge of the values, beliefs, and practices within Mexican-American households in the *colonias*. All of the *promotora*-researchers are native Spanish speakers and reside in area *colonias*. Interview completion was estimated to require a maximum of 20 minutes, a time determined as not burdensome in previous work with these families.

A team of two trained *promotora-*researchers conducted interviews with each mother-child dyad independently, where mothers and children were interviewed separately and had previously provided consent and assent to participate in the study. All interview responses were recorded by the *promotora-*researchers on paper and were then entered into a Microsoft Access database by a research assistant, where all data were double checked for accuracy. All materials and protocols were approved by the Texas A&M University and Baylor University Institutional Review Boards.

Measures were translated into Spanish using a translation-back translation method: 1) translating original English into Spanish, ensuring the English meaning was maintained; 2) back-translating into English by an independent translator who was blinded and not familiar with either the Spanish or English version; 3) comparing the two English versions; and 4) resolving all discrepancies. Interviews were conducted in Spanish.

### Measures

Physical activity neighborhood and household environmental support assessment items were guided and developed using current physical activity environmental literature, previously established environmental assessment instruments, and visual scans of 32 *colonias* conducted by the researchers in 3 areas of Hidalgo County in South Texas, which occurred in February 2011.[39,58-60] Visual scans provided the researchers with an overarching perspective regarding neighborhood and household environmental characteristics applicable for families residing in *colonias*. *Promotora-*researchers reviewed the resulting assessment items and 16 household and 23 neighborhood items were included. Each mother recorded which items existed in her home and immediate neighborhood.

Questionnaires conducted as face-to-face interviews by *promotora*-researchers also included demographic characteristics, health characteristics, barriers to physical activity for children, and open-ended items pertaining to (1) where children currently go to exercise, do physical activity, play sports, or play in a physically active manner; (2) what physical activity options are available for children to do physical activity, exercise, or sports; and (3) how children get to and from these physical activity locations.

Barrier items were developed using current physical activity literature, physical activity barrier scales, and feedback from *promotora*-researchers and included intrapersonal, interpersonal, and environmental barriers. [58,61,62] The final instrument included 28 items. Children were asked to identify which barriers “stop them from doing physical activity, exercise, or more exercise”. Each mother was also asked to identify the barriers that prevent her son or daughter from engaging in exercise, physical activity or sport. Participants were instructed to select all that apply and an open-ended item concluded this portion of the interview allowing participants to include other potential barriers.

Demographic and health information was also obtained and included the following items for mothers and children separately: measured height and weight, general health status, age, gender (child only), birth country, age, number of children living at home (mothers), and household income (mothers). Height and weight were used to calculate body mass index (BMI) for both children and mothers (BMI = weight (lbs)/[height (in)]^2^ × 703), where mothers were considered overweight or obese with a BMI ≥ 25.0 and children were considered overweight or obese with a BMI percentile ≥ 85^th^ percentile using the BMI-for-age percentile growth chart.[63-66] Health was assessed using a single item asking mothers and children separately to self-report their general health given 5 responses: poor, fair, good, very good, or excellent. Mothers and children were also asked to report her/his country of birth.

### Data analysis

Descriptive statistics, including frequencies, means, and standard deviations were calculated to describe the demographic and health characteristics of the sample. Frequencies were also calculated to determine the most commonly reported environmental resources using data collected via the environmental assessments and barriers for childhood physical activity as perceived by *colonias* children and their mothers. Potential differences in barriers for boys and girls, countries of birth, and BMI categories, (1) under to normal weight and (2) overweight or obese, were examined using chi-square tests or fisher’s exact test when estimated cell sizes were less than 5.[67] Analyses were conducted using SPSS v19.

Analysis of open-ended questions followed a deductive approach tailored to identify physical activity opportunities and locations, and available transportation methods. A coding tree was developed based on previous literature within the areas of physical activity barriers, environmental support, rural settings, and Hispanic communities.[33,58,61,62] The coding tree was modified based on feedback from *promotora*-researchers and an initial review of interview responses to ensure saturation of themes. All data were coded independently by two primary coders. When a discrepancy in coding occurred, a third coder was incorporated and consensus was reached. Potential differences in opportunities and locations were examined for gender, birth country, and BMI category as described above.

## Results

### Sample characteristics

The average age of the children was 8.9 years (SD=1.5) and just over half were female (58.5%, n=55). All children and mothers were of Mexican-origin (100%), where 73.4% of the children were born in the U.S. (n=69). Eighty-seven percent of mothers were born in Mexico (n=82). Just under half of the children were either overweight or obese using BMI percentiles (48%, n=45; ≥85^th^ percentile) and 30 of the children were obese (≥95^th^ percentile). Mean BMI percentile for the children was the 68^th^ (SD=32.8) and 12% were either underweight (< 5^th^ percentile, n=5) or at risk for being underweight (5^th^ - 15^th^ percentile, n=6). Mean BMI percentile for children born in Mexico was 62^nd^ (SD=31.6), 70^th^ (SD=33.3) for children born in the U.S., 65^th^ for female children, and 71^st^ for male children. T-tests revealed no significant differences when comparing either set of groups, although greater proportions of male children (53.8%) and children born in the U.S. (52.2%) were overweight or obese as compared to female children (43.6%) and children born in Mexico (36.0%). Eighty-four percent of mothers (n=79) were either overweight or obese (BMI≥25), where 12 were normal weight, 21% (n=20) were overweight, 32% (n=30) were obese class I (BMI = 30.0-34.99), 18% (n=17) were obese class II (BMI = 35.0-39.99), and 13% (n=12) were obese class III (BMI ≥ 40.0).[64] Nineteen (20.2%) mothers reported their health as very good to excellent, with the majority reporting their health as fair or good (n=68, 72.3%). Thirty-seven (39.4%) children reported their health as very good to excellent, with the majority reporting their health as fair or good (n=56, 59.6%). The majority of these children have two married parents, and the majority of families (79%) have 3 or more children living at home. More than half of these families had a total household income less than $699 per month, with 96% of the families reporting a total household income less than $1,500 per month.

### Household and neighborhood environmental resources and supports

Environmental support assessments were completed by each mother pertaining to her household and neighborhood (see Table 1). The mean number of household environmental supports for physical activity was 5.2 (possible range: 0 to 16, SD=2.5). The mean number of neighborhood environmental supports for physical activity was 12.3 (possible range: -2 to 21, SD=3.6), where graffiti and traffic were negatively coded as these hinder activity versus support it. The following physical activity resources were limited in surveyed neighborhoods: recreational buildings (24.5%) and walking tracks or trails (24.5%). Additionally, neighborhoods frequently had the following characteristics shown to hinder physical activity participation: no stoplights (69.1%), traffic (63.8%), and “a lot of graffiti” (47.9%). No significant differences were detected in household or neighborhood environmental assessment items when considering the child’s gender or birth country.

**Table 1:** Environmental assessment: Frequencies for household and neighborhood items (n=94)

**Assessment Item**	**Household Items: n (%)**	**Neighborhood Items: n (%)**
Trampoline	21 (22.3%)	86 (91.5%)
Weight/exercise machine	16 (17.0%)	
Balls (basket, soccer, football)	75 (79.8%)	83 (88.3%)
Basketball hoop/Functionality	26 (27.7%)/11 (11.7%)	79 (84.0%)/56 (59.6%)
Bicycles and/or tricycles	69 (73.4%)	91 (96.8%)
Swing set	17 (18.1%)	82 (87.2%)
Scooter	30 (31.9%)	
Volleyball net	3 (3.2%)	27 (28.7%)
Wagon	14 (14.9%)	
Tire swing	7 (7.4%)	
Tires for children to roll or play with	9 (9.6%)	24 (25.5%)
Push car	48 (51.1%)	
Non-motorized toy car (pedal with feet)	34 (36.2%)	
Yard or outdoor patio space	86 (91.5%)	81 (86.2%)
Paved driveway	14 (14.9%)	
Swimming pool suitable for swimming	19 (20.2%)	59 (62.8%)
A block for walking		54 (57.4%)
Soccer field (formal or informal)		48 (51.1%)
Children playing with balls in streets		86 (91.5%)
Good streets for walking or running		77 (81.9%)
Walking track or trail		23 (24.5%)
Park (in or right next to the *colonia*)		48 (51.1%)
Open space like a field		39 (41.5%)
Paved streets		86 (91.5%)
Children playing games (hide & seek/tag)		72 (76.6%)
Recreational building of any type (in or right next to the *colonia*)		23 (24.5%)
Playground (schools or churches); in or right next to the *colonia*		66 (70.2%)
Traffic		60 (63.8%)
Traffic lights		29 (30.9%)
Lots of graffiti in community		45 (47.9%)

Chi-square analyses examining differences in household and neighborhood resources and supports for normal weight versus overweight-obese children revealed significant differences for the following neighborhood resources: blocks available for walking, soccer fields, volleyball nets, and pools. Children with a normal BMI classification were more likely to have soccer fields within their neighborhood (63.3%) than overweight or obese children (37.8%, χ^2^=6.10, df=1, p=.014). Children with overweight or obese BMI classification were more likely to have blocks for walking (68.9%, χ^2^=4.62, df=1, p=.032), volleyball nets (42.2%, χ^2^=7.683, df=1, p=.006), and pools (75.6%, χ^2^=6.04, df=1, p=.014) within their neighborhoods than children with normal BMI classifications (46.9%, 16.3%, 51.0%). No other significant differences in household or neighborhood resources or supports were detected between BMI classifications.

### Perceived barriers to physical activity

Barriers to children’s physical activity, exercise, or sport reported by children and their mothers were assessed (see Table 2). Subsequently, potential differences in perceived barriers to physical activity, exercise, or sport for boys and girls were examined. The only significantly different (p≤.05) barrier for girls and boys reported by children was unleashed dogs in the street (*χ*^2^=3.77, df=1, p=.05), where more girls (81.8%) perceived dogs in the street to be a barrier to physical activity as compared to boys (64.1%). The only significantly different barrier to physical activity, exercise, or sport for boys and girls as reported by their mothers was time (χ^2^=5.86, df=1, p=.01), where mothers more frequently reported time as a barrier to physical activity for boys (38.4%) than girls (16.4%). Barriers for boys and girls as reported by children and their mothers are presented in Table 2.

**Table 2:** **Physical activity barriers for *colonias *children reported by mother-child dyads: Examining gender differences**

**Barriers**	**Entire Sample**		**Gender**			
	**(n=94)**		**Boys (n=39)**		**Girls (n=55)**	
	**Child**	**Mother**	**Child**	**Mother**	**Child**	**Mother**
Transportation	25.5%	30.9%	23.1%	33.3%	27.3%	29.1%
Dogs in street*^Ch^	74.5%	79.8%	***64.1***%	79.5%	***81.8***%	80.0%
Lack of energy	34.0%	14.9%	35.9%	15.4%	32.7%	14.5%
Traffic	43.6%	40.4%	41.0%	35.9%	45.5%	43.6%
Crime	13.8%	25.5%	12.8%	23.1%	14.5%	27.3%
No motivation	38.3%	13.8%	48.7%	15.4%	30.9%	12.7%
No place like a park	41.5%	38.3%	35.9%	38.5%	45.5%	38.2%
No one to do PA with	25.5%	17.0%	28.2%	23.1%	23.6%	12.7%
Immigration status	1.1%	2.1%	0.0%	5.1%	1.8%	0.0%
Kidnappings	16.0%	23.4%	15.4%	20.5%	16.4%	25.5%
Time*^M^	30.9%	25.5%	33.3%	***38.5***%	29.1%	***16.4***%
Small children at home	1.1%	3.2%	2.6%	0.0%	0.0%	5.5%
No adequate clothing	24.5%	42.6%	28.2%	38.5%	21.8%	45.5%
Can’t leave house alone	1.1%	3.2%	0.0%	0.0%	1.8%	5.5%
No sidewalks	29.8%	48.9%	38.5%	43.6%	23.6%	52.7%
Trash	12.8%	21.3%	17.9%	28.2%	9.1%	16.4%
No place to do PA	34.0%	35.1%	33.3%	35.9%	34.5%	34.5%
No fenced area	35.1%	38.3%	28.2%	41.0%	40.0%	36.4%
No streetlights	44.7%	55.3%	46.2%	53.8%	43.6%	56.4%
Chickens/hens	2.1%	3.2%	2.6%	5.1%	1.8%	1.8%
Gang activity	23.4%	30.9%	28.2%	33.3%	20.0%	29.1%
Cows or goats	2.1%	1.1%	0.0%	2.6%	3.6%	0.0%
Afraid child will get hurt	20.2%	20.2%	20.5%	25.6%	20.0%	16.4%
No friends/family encourage PA	21.3%	6.4%	23.1%	10.3%	20.0%	3.6%
PA is not fun	14.9%	2.1%	20.5%	5.1%	10.9%	0.0%
Asthma	6.4%	7.4%	5.1%	7.7%	7.3%	7.3%
Too hot	55.3%	51.1%	61.5%	51.3%	50.9%	50.9%
Bad weather	51.1%	45.7%	59.0%	41.0%	45.5%	49.1%

*Notes*. ***Bold*** = statistically significant (p≤.05) difference between genders; *^Ch^ = statistically significant difference in barrier for child report; *^M^ = statistically significant difference in barrier for mother report; PA= physical activity, exercise, sport.

Perceived barriers to physical activity, exercise, or sport for children were also examined for potential differences between children born in Mexico and the United States. Two perceived barriers to being active as reported by children significantly differed by birth country: lack of energy (χ^2^=7.37, df=1, p=.007) and transportation (χ^2^=3.75, df=1, p=.05). More children born in the U.S. reported “not having energy” as a barrier to being active (42.0%) as compared to children born in Mexico (12.0%), and more children born in Mexico reported transportation as a barrier to being active (40.0%) as compared to children born in the U.S. (20.3%). Perceived barriers to being active reported by mothers significantly differed by birth country for two barriers: immigration status (χ^2^=5.64, df=1, p=.018) and kidnappings (χ^2^=10.407, df=1, p=.001; see Table 3). More children born in Mexico had a mother that reported immigration status as a barrier to her child being active (8%) as compared to children born in the U.S. (0%), and more children born in the U.S. had a mother that reported kidnappings as a barrier to her child being active (31.9%) as compared to mothers whose child was born in Mexico (0%). Barriers for children born in Mexico and the U.S. as reported by children and their mothers are presented in Table 3.

**Table 3:** **Physical activity barriers for *colonias *children reported by mother-child dyads: Examining differences by birth country**

**Barriers**	**Birth Country**			
	**Mexico (n=25)**		**U.S. (n=69)**	
	**Child**	**Mother**	**Child**	**Mother**
Transportation*^Ch^	***40.0***%	24.0%	***20.3***%	33.3%
Dogs in street	72.0%	80.0%	75.4%	79.7%
Lack of energy*^Ch^	***12.0***%	12.0%	***42.0***%	15.9%
Traffic	44.0%	36.0%	43.5%	42.0%
Crime	20.0%	12.0%	11.6%	30.4%
No motivation	28.0%	16.0%	42.0%	13.0%
No place like a park	36.0%	44.0%	43.5%	36.2%
No one to do PA with	32.0%	24.0%	23.2%	14.5%
Immigration status*^M^	0.0%	***8.0***%	1.4%	***0.0***%
Kidnappings*^M^	20.0%	***0.0***%	14.5%	***31.9***%
Time	24.0%	20.0%	33.3%	27.5%
Small children at home	4.0%	4.0%	0.0%	2.9%
No adequate clothing	32.0%	44.0%	21.7%	42.0%
Can’t leave house alone	0.0%	0.0%	1.4%	4.3%
No sidewalks	40.0%	44.0%	26.1%	50.7%
Trash	20.0%	20.0%	10.1%	21.7%
No place to do PA	40.0%	44.0%	31.9%	31.9%
No fenced area	40.0%	44.0%	33.3%	36.2%
No streetlights	44.0%	44.0%	44.9%	59.4%
Chickens/hens	4.0%	0.0%	1.4%	4.3%
Gang activity	32.0%	36.0%	20.3%	29.0%
Cows or goats	4.0%	0.0%	1.4%	1.4%
Afraid child will get hurt	20.0%	20.0%	20.3%	20.3%
No friends/family encourage PA	24.0%	8.0%	20.3%	5.8%
PA is not fun	16.0%	4.0%	14.5%	1.4%
Asthma	0.0%	4.0%	8.7%	8.7%
Too hot	56.0%	40.0%	55.1%	55.1%
Bad weather	52.0%	40.0%	50.7%	47.8%

*Notes*. ***Bold*** = statistically significant (p≤.05) difference between birth countries; *^Ch^ = statistically significant difference in barrier for child report; *^M^ = statistically significant difference in barrier for mother report; PA= physical activity, exercise, sport.

Chi-square analyses examining differences in barriers to physical activity for normal weight versus overweight-obese children revealed significant differences for transportation, traffic, and crime as reported by children, and transportation as reported by mothers. Children with a normal BMI classification were more likely (38.8%) to report transportation as a barrier to physical activity than children with an overweight or obese BMI classification (11.5%, χ^2^=9.44, df=1, p=.002). Mothers of children with normal BMI classification were more likely (40.8%) to report transportation as a barrier to physical activity than mothers with children who had an overweight or obese BMI classification (20.0%, χ^2^=4.76, df=1, p=.029). Children with normal BMI classification were also more likely to report traffic (53.1%; χ^2^=3.71, df=1, p=.054) and crime (20.4%; χ^2^=3.72, df=1, p=.054) as barriers than children with overweight or obese BMI classifications (33.3%; 6.7%). No other significant differences between BMI classifications were detected.

### Physical activity offerings, locations, and transportation characteristics

Salient themes were identified from open-ended responses describing where *colonias* children currently go to exercise, play sports, or play in a physically active manner as reported by the children and their mothers. Schools were the most frequently reported locations for current physical activities by children (75.5%) and mothers (73.4%), which included school grounds, physical education, and school gyms. Physical education at schools was specifically reported by 12.8% of the children and 11.7% of mothers. The home-area was the second most prominent location for current physical activity (40.4% children, 36.2% mothers) and involved any physical activities occurring within the home, yard, or patio space, and included yard work and recreational activities. Of the children, 18.1% reported parks, 9.6% neighborhood, 5.3% activities with others, and 3.2% reported open fields/lots, sports, walking, and no physical activity. Of the mothers, 25.5% reported parks, 11.7% neighborhood, 8.5% playing, 4.3% exercise equipment, and 3.2% biking. Location codes with a frequency of ≤ 2 for both children and mothers included other homes, running, church, strength activities, cardio activities, and community facilities. No significant differences for locations of current physical activities were detected between genders or birth countries. However, mothers of children with an overweight or obese BMI classification were more likely to report exercise equipment (8.9%) than mothers of children with a normal BMI classification (0%, χ^2^=4.55, df=1, p=.033, fisher’s exact test =.049).

Salient themes identified from the open-ended question inquiring about available physical activity options and opportunities for these children as reported by the children and their mothers were examined. The most frequently reported physical activity opportunities for children were using exercise equipment (67.0% children, 59.6% mothers), which included balls, bikes, trampolines, monkey bars, etc. When a ball was mentioned in relation to a sport itself, the response was double-coded to capture the equipment and the sport. When biking was mentioned, it was double-coded to capture both biking and the equipment. Running (children: 62.8%, mothers: 53.2%), playing (50.0%, 41.5%), sports (26.6%, 21.3%), and biking (18.1%, 24.5%) were the next most commonly reported opportunities reported by both the children and their mothers. Of the children, 17.0% reported cardiovascular activities, 12.8% walking, 8.5% activities at home, 5.3% activities at a park, 5.3% strength activities, 4.3% activities at school, and 3.2% activities with pets and activities at a community facility. Of the mothers, 18.1% reported walking, 14.9% cardiovascular activities, 10.6% activities at a park, 8.5% activities at school, 6.4% none, 4.3% activities at home and activities with music, and 3.2% activities in neighborhood. Opportunity codes with a frequency of ≤ 2 for both children and mothers were activities with others, activities at church, in an open field or lot, and swimming.

Significant differences for birth country, gender, and BMI classification for physical activity opportunities were detected. More boys (38.5%) than girls (18.2%) reported “sports” (χ^2^=4.807, df=1, p=.028). Similarly, more mothers of boy children (41.0%) reported “sports” as a physical activity opportunity as compared to mothers of girls (9.1%; χ^2^=13.413, df=1, p≤.0001). More children born in Mexico (32%) reported biking as a physical activity opportunity as compared to children born in the U.S. (13%, χ^2^=4.45, df=1, p=.035). Overweight or obese children (20.0%) and mothers of these children (28.9%) were more likely to report walking as an opportunity as compared to children with a normal BMI classification (6.1%, χ^2^=4.06, df=1, p=.044) and their mothers (8.2%, χ^2^=6.80, df=1, p=.009). Normal weight children (10.2%) were more likely to report strength training activities as opportunities as compared to overweight or obese children (0%, χ^2^=4.85, df=1, p=.028, fisher’s exact test = .057). No other significant differences between groups were detected for children or mothers’ responses.

When the children were asked where physical activity opportunities were located, 31% said they were at a school, 25% said their home (house, patio, yard, etc.…), 14% said a park (various city parks were mentioned), and 8% said in their neighborhood (the streets outside of home, in the *colonia* or neighborhood). Forty-four percent said these were located within their same town, 7% said a neighboring town within 5 miles, 12% said a neighboring town 5–10 miles away, and only one person said a town 10–15 miles away. When asked how they get to and from the physical activity opportunities, 47% of the children stated they use the school bus for transportation to physical activity options, 45% are driven in a car, and 20% said they walk. No children mentioned using any type of public transportation or cycling. Three-fourths of these children’s mothers owned a car and 60% of them reported having a car available to them during the day. Alternative forms of transportation included relatives (41%) and family friends (20%); although, 54% of mothers said they are charged to use these alternative forms of transportation.

## Discussion

This study furthers our understanding of the physical activity environment for high-risk, underserved Mexican-origin mothers and children living in Texas border *colonias* by describing (1) household and neighborhood infrastructure for physical activity, (2) perceived barriers to physical activity for these children, taking into account potential gender and birth country differences, and (3) physical activity opportunities and locations. Specifically, environmental support was assessed using items derived from current literature and previously developed environmental audits for rural and urban settings.[39,58-60] Given the uniqueness of *colonia* areas as compared to both urban and rural areas, it was imperative to tailor environmental support items to be culturally relevant and specific. In these assessments, several environmental factors consistently supportive of physical activity and health in previous research were reported. These included paved streets, streets deemed good for walking or running,[68] playgrounds (church or school) near the *colonias*,[69,70] and supportive social norms of play via active children in the neighborhood (children playing with balls in the street and children playing games in neighborhood).[71] These assessments also revealed several environmental traits unsupportive of physical activity within *colonias* households and neighborhoods that are consistent with previous research and often more salient for low-income children. These included few recreation buildings[72,73] and traffic lights,[74,75] limited parks and open spaces (fields),[74] and high levels of traffic[73,76] and graffiti.[72,77]

Environmental characteristics unique to *colonias* were also identified in this study, including a high occurrence of yard and/or patio space and exercise equipment (e.g., balls, bicycles/tricycles, etc.…). Although relationships between yard presence and/or yard size and exercise equipment with physical activity are not always supported in the literature,[71,78,79] the home area, including yards and patios, was reported as the second most common location for physical activity among participating children. While many *colonia* houses are on relatively generous lots, often fenced or walled-in, the houses themselves are usually built piece-by-piece as the family can afford to do so (also referred to as self-built or incremental construction), usually providing limited space for active recreational endeavors indoors.[10,24] In addition, several neighborhood barriers to physical activity were highlighted within this study, including unleashed dogs and traffic for all children and kidnappings for U.S.-born children. These distinct contextual characteristics provide insight into why confining children to the home-space or yard could be perceived as safe and more supportive of physical activity. This explanation also provides insight into another potential household environmental barrier to physical activity distinct for *colonias* children, as relatively few driveways were paved (15%). While paved driveways are not usually included as physical activity resources in the literature, given the context of *colonia* housing and neighborhood barriers, paved driveways could provide an additional home-based physical activity resource for children. Future research should investigate benefits and safety issues around paved driveways as a potential physical activity resource among less developed and unincorporated areas.

Items representing perceived barriers, both environmental and individual-level, were also derived from current literature and tailored for cultural relevance. As with the environmental assessment, interview responses highlighted barriers that are both unique to this population and similar to those within other samples. Unleashed dogs in the *colonia* streets was the predominant barrier to children being physically active for the sample as reported by both children and their mothers, regardless of gender or birth country. While the intensity of this finding is somewhat distinct to the *colonias* population as compared with other literature, unleashed dogs have been reported as relevant barriers to physical activity for other populations living in communities with potentially less infrastructure and development, such as rural U.S. areas, and for some international adolescents.[77,80,81] These findings support the need to identify whether unleashed dogs are an actual or perceived environmental barrier to physical activity. Future initiatives should address the perceived barrier of unleashed dogs within these neighborhoods and educate on dog protection. Additionally, initiatives need to acknowledge the intensity of this perception/fear, while also investigating actual safety threats of unleashed dogs, as incongruence between perceptions and actual threats would suggest different intervention foci. Regardless, initiatives encouraging physical activity of *colonias* children, especially girls, should address this barrier with children themselves and their mothers.

Traffic, lack of street lights, and bad weather/heat are other environmental barriers prominent in this study previously identified for children and other underserved communities.[62,76,80,82,83] Although heat is somewhat unavoidable during most of the year in south Texas, future education and programming should address risks associated with heat. Concerns about traffic and lack of streetlights should be communicated to policy makers and non-governmental organizations to help increase advocacy for change and inform about infrastructure and zoning needs. One challenge to making environmental changes is the fact that many *colonias* are not within local governance, but rather within county-level governance. Advocacy efforts should occur at the local, county, and state-level, and communication across all levels is essential.

Several group differences detected in this study highlight unique considerations when encouraging physical activity among *colonias* children. Time was reported by mothers as a significantly more prominent barrier to physical activity for boys in comparison (39%) to girls (16%). While time is not the predominant barrier for boys in this study, this difference is worth examining and is consistent with previous research reporting time and other priorities (work, school, family) as barriers to physical activity for Hispanic adolescent boys.[83] Because low income families are more likely to rely on children for household chores and babysitting,[84] boys’ perception of free time available for physical activity could decrease. In addition, mothers and fathers tend to lack the time to supervise or provide transportation at the appropriate times for boy’s physical activities, notably sports participation.[85] Boys participate in sports activities significantly more often than girls,[86] thus linking less time available for boys to participate in their reported physical activities than their female counterparts. Another potential explanation of this gender difference can be seen through the lens of Hispanic adult males where differences between the American culture and the culture of their countries of origin have been identified as barriers to physical activity. Specifically, in the American culture Hispanic men reported having less time available and fewer social activities involving physical activity (e.g., soccer) as compared to their home country’s culture.[83] This difference could also be important for Hispanic boys, suggesting that time should be considered as a barrier when planning physical activity initiatives that include Mexican-origin boys. Future research should also further examine this finding.

Four differences in barriers were also detected when comparing children born in different countries. Children born in Mexico were more likely to report lack of transportation as a barrier to physical activity (42%) than children born in the U.S. (20%), even with no significant differences between car ownership or availability during the day and mother’s employment (part/full-time versus not working or full-time versus part-time/not working) for mothers of children born in Mexico (car ownership: 75%, car availability: 50%, any work: 10%, full-time: 10%) as compared to the U.S. (car ownership: 81%, car availability: 65%, any work: 24%, full-time: 6%). Physical activity initiatives targeting children residing in *colonias* should take this into account, as up to 1/3 of colonias residents are born in Mexico.[14]

Children born in the U.S. were more likely to report a lack of energy (42%, n=29) as a barrier as compared to children born in Mexico (12%, n=3). Although the subsample of children born in Mexico is modest, this difference is notable. While lack of energy, feeling tired, or fatigue has been inversely related with physical activity and positively related with sedentary time among adult populations,[62,83,87] it is not usually examined as a potential barrier of physical activity for children[71,83,88] and distinctions between first and second generation Mexican-origin children have not been examined. However, food insecurity is related with fatigue among other health risks and obesity for children, where children of low income families and specifically *colonias* families are at greater risk for food insecurity.[56,89] Although our findings do not clearly support the explanation or provide insight into why American-born versus Mexican-origin children were more likely to report energy as a barrier to physical activity, 96% of the study population has a monthly income < $1,500, suggesting all families in this study have a heightened risk for food insecurity. The risk for food insecurity and associated fatigue is even greater when considering all Mexican-born children and approximately half of U.S.-born children reporting “lack of energy” as a barrier came from families making < $700 per month. Another potential explanation could exist when considering the south Texas climate. Given extreme high temperatures and many families lacking air conditioning, a lack of energy could be explained given the heat and potential dehydration. In considering this potential explanation and the high frequency of participants reporting heat as a barrier to physical activity in this study, physical activity initiatives must take into account the environmental element of heat.

Differences seen between normal weight children and those who were overweight-to-obese suggest cultural preferences and support metabolic understandings.[4,90] Given that children with normal BMIs were more likely to have soccer fields available in their neighborhoods and report strength training as an available physical activity opportunity than overweight/obese children suggests that normal weight children either have more resources supportive of vigorous and strength activities, both related with greater metabolic expenditure than light-to-moderate activity,[4] or are more likely to be aware of these resources as compared to overweight-to-obese children. This is also supported when considering that overweight-to-obese children were more likely to have blocks for walking and report walking as an available physical activity opportunity than normal weight children. Future research needs to understand whether there is a lack of resources or an incongruence between perceptions and availability. Outcomes of this assessment would inform the direction of future initiatives. The greater reports of soccer fields within neighborhoods of children with normal BMIs also lends insight into how these children might be or could overcome their reported barriers of transportation, traffic, and crime; as they would be able to walk to soccer fields within their neighborhoods and would most likely be associated with larger groups of people while there, which could reduce perceptions of fear.

Locations of current physical activities and available physical activity opportunities emphasize the importance of school and home environments and supportive physical activity resources and modes including exercise equipment, running, playing, sports, and biking. Although most physical activity opportunities reported are located within a relatively short distance from the areas that the children live (usually in the child’s “home town” or a neighboring town of the *colonia* in which they reside), only 20% of these children reported walking as a form of transportation to these locations. In addition, this supports both the need for school-based physical activity opportunities that incorporate transportation and the importance of home-based physical activity options. These findings substantiate previous studies that suggest the greatest opportunity for lower income children to engage in physical activity is within the school setting, as school-based physical activity has demonstrated an equalizing effect for socio-economic status differences associated with physical activity.[91-93] These results also support the continued call for physical education programs and free-play/recess during the school day and the utilization of school transportation options within after-school programming to allow for additional physical activity opportunities and engagement within high-risk, underserved populations.[92-95]

Parks and churches are also environmental structures that support physical activity.[69,70,96] However, a limited number of parks are documented within or near the *colonias*. With 42% of children reporting a barrier of “no place like a park,” and research supporting the relationship between parks and playgrounds with physical activity for children, physical activity programming for children living near parks and future policies for *colonias*-area planning to include more park spaces and/or playgrounds should be considered.[70,96] Additionally, future research needs to further examine the quality and availability of playground and park features in *colonias*, as some research suggests it is not simply the presence of a park or playground that is related to physical activity, but rather the features and quality of the facilities.[97,98] In this study, one child and one mother reported a church as a location for physical activity. Given the strong religious heritage of the Mexican culture,[99,100] churches could potentially support physical activity of *colonias* residents. In addition, previously reported relationships between church facilities and physical activity for adolescents, including rural and minority youth, [69] support future research aimed at examining the current role and future potential of churches within *colonias* communities in regards to policies and programming related with physical activity.

The use of mixed-methods and mother-child dyads to examine environmental supports, barriers, and locations of physical activity are strengths of this study design, in addition to the examination of differences by gender and birth country. Another strength lies in the tailoring of environmental measures (assessment and barriers), as these items were both based on previously used measures and culturally tailored for relevance and specificity to this distinct population. However, given this approach, validity and reliability of these measures is not established. Additionally, although these results provide a rich description of the physical activity environment for children residing in south Texas colonias, causality was not examined. While future studies should seek to validate these measures in similar *colonias* and new-immigrant populations and examine causal relationships, current research supports the need to describe the environmental landscape and understand environmental factors of physical activity and obesity to inform physical activity and obesity-prevention policies and initiatives, especially among our highest risk and underserved populations.

## Conclusions

While this study focuses on children residing in *colonias* within the Texas-Mexico border region, these findings should be used to inform future research, policy, and public health initiatives for other *colonias* communities along the U.S.-Mexico border and rapidly growing new-immigrant destination communities seen throughout America. While immigrants in the past century have primarily settled in one of a few gate-way urban cores, more recent trends evidence an increase of new-immigrant destinations throughout rural and less-settled urban-periphery counties found across the U.S., including the Midwest and Southeast among others.[9,101] Recent immigration trends reveal approximately half of immigrants residing in urban areas and 2/3 of immigrants in rural areas are Hispanic, where 48% of rural immigrants are of Mexican-descent.[9] Given most Hispanic and specifically Mexican immigrants are faced with similar challenges as *colonias* residents regardless of geographical location, including severe poverty, educational disadvantages, escalated risk for chronic diseases, underemployment, and residences within ethnic enclaves,[9]*colonias* can serve as an archetypal example for researchers, public health practitioners, and policy makers in understanding these new-immigrant destinations scattered across the nation.

Future research should aim to understand the social environment of children residing in *colonias*, in addition to school and church-based policies, programming, and infrastructure as they relate to physical activity support. Future physical activity initiatives targeting *colonias* children should incorporate a community-based participatory research (CBPR) approach working with community *promotoras* and consider programs incorporating currently available exercise equipment, while developing physical activity initiatives within schools, homes, and parks that incorporate transportation for children. Policy makers should use these findings to help guide decisions about resource allocation within *colonias* and new-destination immigrant communities to increase parks, playgrounds, and access to school grounds. This study suggests that environmental resources and barriers for *colonias* children are both similar to previous studies and distinctively unique. As expected, built resources for these communities are limited; however, knowledge of barriers and available physical activity resources within *colonia* households and neighborhoods offers insight and can help guide policy and physical activity initiatives. Future work should also focus on facilitating neighborhood groups and linkages with non-governmental organizations to increase advocacy for community change.

## Competing interests

The authors declare that they have no competing interests.

## Authors’ contributions

MRUM contributed to the conception of this study, analysis and interpretation of the data, and drafted the manuscript. JRS contributed to the conception and design of the study, acquisition of data, and critically revised the manuscript. MSP contributed to the analysis and interpretation of data and critically revised the manuscript. WRD contributed to the design of the study and critically revised the manuscript. All authors have given final approval of the manuscript for publication.

## Pre-publication history

The pre-publication history for this paper can be accessed here:

http://www.biomedcentral.com/1471-2458/13/14/prepub
